# Evaluation of the Acaricidal Effectiveness of Fipronil and Phoxim in Field Populations of *Dermanyssus gallinae* (De Geer, 1778) from Ornamental Poultry Farms in Italy

**DOI:** 10.3390/vetsci9090486

**Published:** 2022-09-08

**Authors:** Alessandro Guerrini, Benedetto Morandi, Paola Roncada, Gianfranco Brambilla, Filippo Maria Dini, Roberta Galuppi

**Affiliations:** 1 Department of Veterinary Medical Sciences, University of Bologna, Via Tolara di Sopra 50, Ozzano dell’Emilia, 40064 Bologna, Italy; 2 Istituto Zooprofilattico Sperimentale dell’Umbria e delle Marche “Togo Rosati”, Via Salvemini 1, 06126 Perugia, Italy; 3 Istituto Superiore di Sanità, Dipartimento Sicurezza Alimentare, Nutrizione e Sanità Pubblica Veterinaria, Reparto Malattie Trasmissibili con gli Alimenti, Viale Regina Elena, 299, 00161 Roma, Italy

**Keywords:** phoxim, fipronil, *Dermanyssus gallinae*, resistance, pharmacovigilance

## Abstract

**Simple Summary:**

The poultry red mite *Dermanyssus gallinae* is a blood-sucking ectoparasite responsible for serious animal health and welfare concerns in egg-laying hen facilities, with impacts on productivity and public health. Traditionally, its control is based on the use of synthetic acaricides. Their extensive use has resulted in the development of acaricide resistance. While industrial farms are under strict legislative control, amateur breeders tend to use cheaper pesticides such as phoxim (licensed in poultry) but potentially also unauthorized pesticides, such as fipronil. The aim of this study was to evaluate the in vitro acaricidal activity of different concentration of these two molecules on field populations of *D. gallinae*, collected from ornamental chicken farms in Italy. Their effectiveness was significantly associated with the dose used, but a great variability of lethality rate was observed for fipronil with the increase in dilution. For phoxim, some outliers, with apparently lower sensitivity, were observed particularly in one farm, suggesting that a resistance phenomenon was triggered in this mite population. For this reason, it is necessary to underline the importance of the utilization of authorized products at correct dosages and times of treatment and the need for alternative molecules to avoid the onset of drug resistance phenomena.

**Abstract:**

The poultry red mite *Dermanyssus gallinae* is the most important blood-sucking ectoparasite in egg-laying hen facilities. The aim of this study was to evaluate the in vitro acaricidal activity of different concentration of authorized (phoxim, ByeMite^®^, 500 mg/mL) and unauthorized (fipronil, Frontline^®^ 250 mg/100 mL spray) molecules on 14 field isolates of *D. gallinae* collected from different ornamental poultry farms from different Italian regions. The sensitivity test was performed by contact exposure to four concentrations of each insecticide diluted at 1:5 (10,000-2000-400-80 ppm for phoxim, 500-100-20-4 ppm for fipronil) on a filter paper. The effectiveness of the treatment was significantly (*p* < 0.0001) associated with the dose of the pesticide used. Considering the mean lethality, phoxim has greater efficacy compared to fipronil (*p* < 0.001). A great variability of lethality rate was observed with the increase in fipronil dilution; conversely, for phoxim, some outliers were observed, particularly in one farm, suggesting the hypothesis that a certain degree of resistance in the mite population could occur possibly as a consequence of the continual contact with the molecule. This underlines the importance of the use of licensed products administered at correct dosages and the need for alternative molecules to avoid the onset of drug resistance phenomena.

## 1. Introduction

The poultry red mite *Dermanyssus gallinae* (De Geer, 1778) is the most important cosmopolitan blood-sucking ectoparasite in egg-laying hen facilities [1]. This mite poses serious animal health and welfare concerns, adversely affecting productivity and impacting public health [2]. The red mite is a nocturnal obligate ectoparasite with a high proliferation rate, causing severe stress, irritation, eggs quality and quantity losses [3,4], anemia, and, in the most serious cases, may lead to death [5]. In addition, it may act as a vector of pathogens, including *Salmonella gallinarum* and *Salmonella enteritidis* [6], *Erysipelothrix rhusiopathiae*, *Pasteurella multocida*, *Borrelia anserina*, *Chlamydia* spp., *Coxiella burnetii*, avian poxvirus, Newcastle Disease virus, and St. Louis encephalitis virus [6,7]. In the absence of avian hosts, *D. gallinae* may infest mammals [8,9], including humans, where it can cause a typical dermatitis known as gamasoidosis or dermanyssosis, especially in workers that work in highly infested egg-laying hen aviaries [10,11,12]. This problem can commonly occur for amateur breeders as well [13]. Less intensive farming systems such as barns and free-range and organic farms show higher prevalence rates than cage systems [14]. However, in Italy, the percentage of infested poultry industrial farms reaches 90% [3]. Anyway, few data are available on the infestation rate in backyard poultry farms. The red mite is almost ubiquitous and challenging to eradicate in rural or in ornamental breeding [15] due to the multitude of farmed species present, potential contacts with wild hosts, and un modern breeding structures, which provide more hiding places for mites. The presence and distribution of *D. gallinae* are not only greatly influenced by the conditions of the rearing environment but also by the efficiency of products and methods used for its control. Traditionally, the control of *D. gallinae* is based on the use of both synthetic acaricides, such as organophosphates, carbamates, pyrethroids and spinosyns [16,17], and natural acaricides such as essential oils [14,18]. The efficacy of synthetic molecules is well-documented, showing their acaricidal effect at each mite’s stage level. However, increasing legal restraints on the use of many active compounds and the scarcity of authorized products force farmers, especially rural farmers, to use illegal or off-label products on free sale for agricultural use or products that are registered for other farm-animal species, with the risk of residues in the food chain [2,3]. The extensive use of synthetic chemicals and the inherent features of the life history of red mites (short life-cycle and high fecundity) resulted in the development of acaricide resistance [19,20]. Drug resistance phenomena relative to molecules approved and unapproved for use on poultry, including alpha-cypermethrin, bifentrin, carbamates, carbaryl, cypermethrin, deltamethrin, dichlorodiphenylitricloroethane (DDT), dichlorvos, phenitrothion, fipronil, flumethrin, flurathiocarb, permathion, phenothrin, tetramethrin, and trichlorfon, have been reported by many countries, including Czechoslovakia, Korea, France, Serbia, Italy, and Sweden [21,22]. Furthermore, the indiscriminate use in poultry promoted the presence of residues in eggs [23,24,25] and the environment, with possible and continued pesticide exposure relative to the workers [3]. 

In 2017, the isoxazolinic compound fluralaner was approved for the control of *D. gallinae* in egg-laying hens by administration via drinking water and with a withdrawal period of 0-day for eggs relative to human consumption [26]. This next-generation product, already known as acaricide in dogs, has revolutionised the control plans of the parasite in breeding, providing valid and irreplaceable assistance. However, its use is prohibitive for many amateur breeders, both in terms of quantity and cost, without neglecting complications deriving from its veterinary medical prescription. Therefore, farmers in this category tend to turn to cheaper pesticides such as phoxim (licensed in poultry) for applications in poultry houses and in the presence of the animals, but these are potentially unauthorized, including easily accessible molecules that are on free sale, such as fipronil. In fact, the use of fipronil in food-producing animals, including poultry, is not only prohibited in Europe, but also in the United States or any other country [27]. However, this pesticide remains easily accessible for applications on other animal species (dogs and cats), increasing the risk of illicit use on farms. In fact, in these farms, the presence of fipronil was recently detected in eggs and in feathers, confirming that the use of fipronil as a pesticide may be common [28]. 

Given that an extensive and incorrect application of pesticides against *D. gallinae* could promote the selection of resistant mite populations, the aim of this study was to evaluate the in vitro acaricidal activity of different concentrations of fipronil and phoxim on field populations of *D. gallinae*, collected from ornamental chicken farms in Italy.

## 2. Materials and Methods

### 2.1. Ornamental Poultry Farms Tested

In the ornamental poultry farms selected for the study, fewer than 250 ornamentals pure breeds chickens were raised (between 40 and 200 chickens) and raised with the free-range method for beauty competitions or for the self-consumption of meat and eggs. Chicken houses were represented by wooden and/or metal structures, with nests for eggs’ deposition and perches for resting at night. With this method of breeding, during the day, animals usually have paddocks for scratching at their disposal. These breeds are not subject to light or temperature conditioning and they preserve their natural biological reproductive cycle. Subjects that do not respect the expected breed standard and are not suitable for beauty competitions are intended for self-consumption, while eggs are incubated, used for home consumption, or sold. 

The farms selected for this study were involved thanks to the collaboration of the farmers, who had been asked if the presence of *D. gallinae* was common in their farm and, in a second step, asked what products were used and the periodicity of treatments against *D. gallinae*. In all farms considered in this study, no chemical acaricidal treatments were used for at least 3 months prior to mite collection. The characteristics of the ornamental poultry farms, the breeds raised, and the pesticides used (if known) for treatments against *D. gallinae* are summarized in Table 1.

### 2.2. Populations of Dermanyssus gallinae Tested

**Figure 1:**
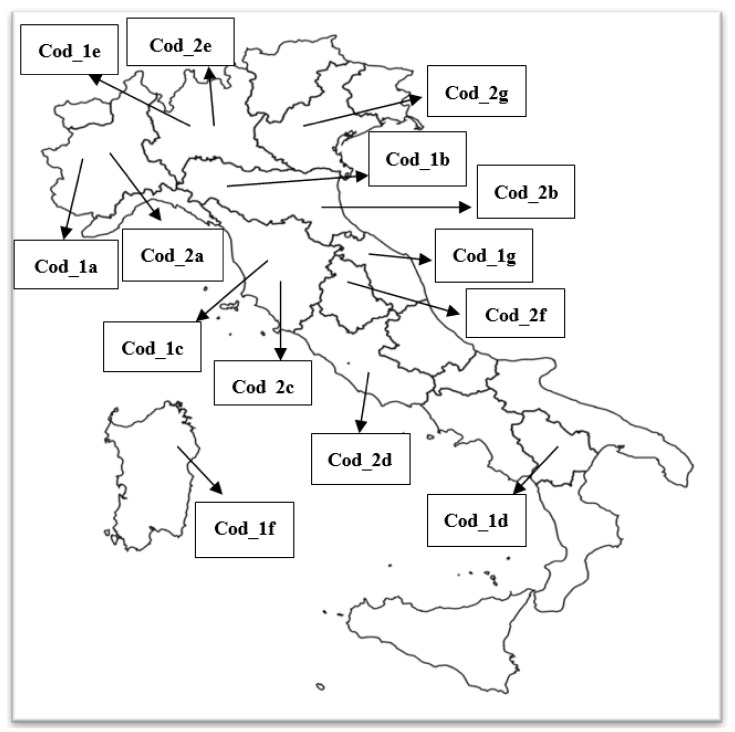
Location of the farms from which the different field population of *Dermanyssus gallinae* tested were sampled.

A total of 14 field populations of *D. gallinae* were tested, each of which was collected from one of the ornamental poultry farms described above and that were located in different Italian regions (Figure 1); each one identified with a unique code. The samplings were carried out between July and September 2020. The mites, both adults and juvenile stages, were collected by the farmers from 4 to 5 different nests of mites located in different points of the poultry houses (in nests, perches, and ravines) or directly on the chickens at night. During mites’ feeding, using small brushes, directly place 150 mL airtight containers. The containers were subsequently sent to the laboratory. The identification of the mites was performed morphologically, according to the key suggested by Di Palma et al. [29]. For 2 mites samples (Cod_1f and Cod_2d) from 2 different region, Sardinia and Lazio, the susceptibility test was not immediately performed, and mites were maintained in vitro for a maximum of 3 days, following the method described by Nunn et al. [30].

### 2.3. Products Tested

Commercial solutions of fipronil and phoxim were used and diluted to produce a range of 4 concentrations. In particular for fipronil, starting from the commercial product purchased at a pet store, at a concentration of 250 mg/100 mL (Frontline^®^ spray, Merial Italia S.p.A.), 1:5 serial dilutions were made obtaining 4 final concentrations: 500, 100, 20, and 4 ppm. For phoxim solutions, from commercial products purchased with a veterinary prescription, at a concentration of 500 mg/mL (ByeMite^®^, Bayer S.p.A.), serial 1:5 dilutions were carried out starting from a dilution of 10 mg/mL (concentration used 10,000, 2000, 400, and 80 ppm). According to the manufacturer’s instructions, the concentration considered effective for causing mites’ death is 2000 ppm. For dilutions of both products, sterile distilled water was used. The water was also used as a negative control.

### 2.4. Sensitivity Assay 

The test was performed as phenotypic assay by evaluating the acaricidal effect by contact exposure to different concentrations of the products distributed on a filter paper [31]. Petri dishes (6 cm Ø) were used, and a filter paper disc of the same diameter was placed on the bottom. Two-hundred microliters of each dilution of the products tested were pipetted on each filter with a spiral motion to ensure the uniform distribution of the pesticide. Each test was carried out in triplicate; i.e., for each mite sample, 3 plates were prepared for each dilution of each molecule to be tested and for the negative control. Each plate was identified with a date, product, and dilution tested. Subsequently, a maximum number of 20–85 mites [1,32], taken directly from the airtight containers, were placed onto the filter paper in each plate. The mites introduced included both adults and nymph. Immediately after the introduction of mites, the Petri dishes were hermetically sealed with a membrane (Parafilm^®^) to prevent mites from escaping and incubated at 27 ± 2 °C for 24 h, keeping them upside down to prevent condensation on the lid. The reading of the plates and the relative mite count was carried out macroscopically using a magnifying glass. For each plate, including the control, the total number of live and dead mites present was counted, and mites that did not show any movement for a continuous observation of a few seconds were considered dead. If present, moribund mites were counted as dead. Nymphal and adult stages were not differentiated.

### 2.5. Statistic Analysis

Data collected during the study were processed using STATA 15 (StataCorp LLC, College Station, TA, USA) software. Farm of origin, the active compound tested and its dilution, the number of live and dead mites and the total mites counted for each plate, and the lethality rate computed as the number of dead mites/total number of counted mites were the grouped variables. The non-parametric Mann–Whitney test was used to evaluate the lethality rate distribution at the different treatment and dilution. The Pearson’s χ^2^ test was used to compare lethality rates and the categorical variables. The results were considered to be significant when *p* ≤ 0.05. Raw data are available in Table S1.

## 3. Results

**Figure 2:**
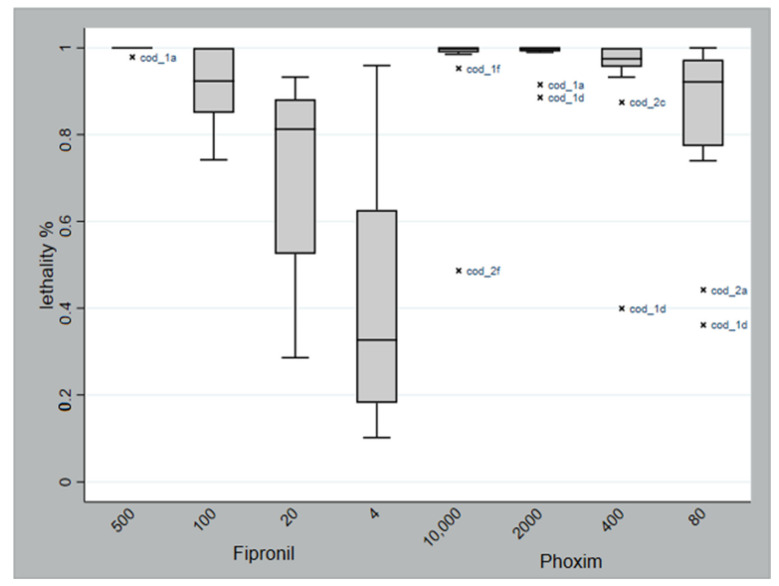
Boxplot of the average lethality for the mites coming from the different farms at different treatment concentration (ppm) for the two tested products. x cod_outliers representing farms considered as potential critical farms for the presence of a lower efficacy of acaricides.

On average the number of mites present in plates does not change in relation to the treatment received, indicating a homogeneous distribution within the plates (*p* = 0.791). Physiological mortality, deduced from the mean lethality of the control plates, was 22.2%, while the mean post-treatment lethality was 77.3% for fipronil and 92.7% for phoxim. Table 2 shows the average lethality related to the concentration of the pesticide to which the mites were exposed. Comparing the physiological mortality with the ones obtained at the minimum dosage used for each product, the difference was significant (χ^2^ = 119; *p* < 0.0001 for fipronil and χ^2^ = 844; *p* < 0.0001 for phoxim); i.e., the effect due to the molecule is still present even at the lowest dosage. The ratio of dead to alive mites at the different tested concentrations, considering the treatment with fipronil, shows a significant difference (χ^2^ = 1200; *p* < 0.0001); the same applies to phoxim (χ^2^ = 248.54; *p* < 0.0001); thus the effectiveness of the treatment is significantly associated with the dose of the pesticide used. Considering the mean lethality as a continuous variable, it appeared that phoxim has greater efficacy compared to fipronil (*p* < 0.001). While fipronil and phoxim do not significantly differ in the first dilutions (*p* = 0.148), the other three dilutions significantly differed to each other (Table 3). The mean lethality of the mites collected from the different farms at different treatment dosages of the two tested products is graphically represented by a boxplot (Figure 2). A great variability in lethality rates in different mite populations can be noted with the increase in fipronil dilution, whereas this variability is considerably lower for phoxim. However, for the latter, there were some outliers, particularly for mites from the farm “Cod_1d”, evident for concentrations at 2000, 400, and 80 ppm.

## 4. Discussion

The poultry red mite *D. gallinae* is a major concern in poultry farms such that a great variety of acaricides, lawful or illegal, are used by farmers throughout Europe, and several cases of finding residues of acaricides in eggs have been reported by the national and international press, with particular reference to fipronil [33,34]. As previously mentioned, the indiscriminate and incorrect use of acaricidal products can stimulate the selection of resistant mite populations. In the present study, the in vitro acaricidal efficacy of two commercial products (one authorized for use in chickens and the other is unauthorized) was tested against different field populations of poultry red mites from various ornamental chicken farms. In the tested farms, the anamnesis reports the occasional use of phoxim, but the possible illicit use of fipronil is unknown. The active compounds (phoxim and fipronil) were tested at different dilution with a 1:5 ratio. In particular, the product containing phoxim is a concentrated emulsion, and according to the manufacturer, 2000 ppm is indicated as the used concentration against *D. gallinae*, while for fipronil, unauthorized for the scope, the series started from a 1:5 dilution of the commercial product. Lethality rate and, consequently, the effectiveness of the treatment were significantly associated with the concentration of the active ingredient used. For fipronil, the highest lethality was found at the highest concentration tested, while when increasing the dilution of the product, there was a significant decrease in lethality, which is associated with an increasing variability of effectiveness on the different populations of mites from different farms. The use of fipronil, a progenitor of the phenylpyrazole class, against *Dermanyssus* in addition to being prohibited is rather empirical. In fact, even if used as an insecticide in agriculture and against ectoparasites, including ticks, in pets such as dog and cats [35,36], there is little evidence in the literature regarding its in vitro effectiveness against *Dermanyssus*, and there are conflicting data. In an in vitro study, Kim et al. [37] tested fipronil as a pure substance by calculating how many milligrams of molecule were present in one cm^2^ of filter paper in contact with the mites. They found that fipronil was active with an LC_50_ at >5 mg/cm^2^. Another more recent study conducted by Wang et al. [31] showed an LC_50_ between 0.17 and 2.10 µg/cm^2^. With the maximum dose used in the present study (500 ppm), we tested the efficacy of 7.6 µg/cm^2^ (0.007 mg/cm^2^) and obtained 99.8% mortality. By comparing these data, it is therefore not surprising that we found a wide range of inefficacy at lower dosages. Probably, there may be a variable efficacy of the product on different mite populations or this may be due to a different intrinsic sensitivity of the mites or due to resistance phenomena for an illegal use of fipronil or, in any case, for its substantial diffusion in the environment. Theoretically speaking, being an unusable molecule in poultry farming, it should have never met field mites’ populations, resulting in a development in resistance. Nevertheless, in some of the same farm types, the presence of fipronil was detected in eggs and feathers, thus confirming that the use of fipronil as a pesticide may be common [28]. The pharma-toxicological profile of phoxim, in relation to *D. gallinae* mites tested, resulted in adequate control (i.e., very high lethality was detected), exerting an acaricidal effect consistent with the concentrations indicated by the manufacturer (2000 ppm). This is in agreement with Zdybel et al. [32] who, testing phoxim-based products, showed lethality rates of 95–100% at concentrations ranging from 4000 to 6000 ppm. For phoxim, in the present study, the decrease in efficacy with increasing dilutions was present but to a lesser extent compared to fipronil. This behavior has also been shown for another poultry hematophagus ectoparasite, *Ornithonyssus sylviarum*, when exposed to decreasing concentrations of phoxim [38]. Moreover, contrary to what observed for fipronil, in phoxim, there is a poor variability of response between mites coming from different farms at decreasing concentrations. It should be emphasized that for six mite populations, highlighted as outliers in Figure 2, the lethality rate at some concentrations was decidedly lower than the others. The outliers can be interpreted as a decrease in the effectiveness of the treatment in some field isolates; it may be due to various reasons, such as possible resistance to the active ingredient as a result of its incorrect use (dosage and treatment periodicity). An interpretation of this type could be applied, in particular, to the pattern of the mite coming from farm “Cod_1d” (Figure 1). In this farm, located in the Basilicata region, the presence of *Dermanyssus* was reported as constant over time despite the implementation of monthly treatments with phoxim. This supports the hypothesis that a certain degree of resistance to the product could be established in this population of mites acquired generation after generation, as is already observed in populations of other species, such as *Tetranychus* *cinnabarinus* [39]. Moreover, it is crucial to associate in vitro evaluations to molecular tests in order to identify resistance genes or their alterations. However, our method used for the evaluation of in vitro acaricidal activity is fast and easy to apply, allowing the obtainment of rapid preliminary results for highlighting potential new resistances. The situation evidenced in our study points to a hypothetical use–misuse of these molecules and the need for a radically different approach to control *D. gallinae*. One of these could be the use of organic pesticides that are generally highly appreciated by breeders, both amateur and not, who favorably view the acaricidal effect associated with a poor environmental impact and, possibly, result in eggs and meat without residue. This could be the case of the use of the phytoextracts with acaricidal activities. As an example, the use of different plant extracts, essential oils, and related compounds derived from plants such as *Eugenia caryophyllata*, *Cinnamomum camphora*, *Asarum heterotropoides*, and *Cnidium officinale* or some vegetable oils, including Neem oil, *Cassia* and *Cinnamon* essential oil, containing the active ingredient cinnamaldehyde, were effective against *D. gallinae* [40,41]. However, efficacy, low toxicity, and the absence of residues in food of natural products must be further tested. This type of approach and choices will be the research area of the future.

## 5. Conclusions

Although this is a preliminary study, the results obtained, particularly the presence of outliers observed in phoxim, focus our attention on the possible emergence of resistance spots in natural populations of *Dermanyssus* to molecules belonging to the organophosphate family, which are often used in farms. On the other hand, the considerable variabilities in responses to fipronil, particularly at the lowest concentrations, suggest a widespread decrease in susceptibility underlining the possible adaptation of different mite populations for the illicit use of the molecule. This points out the importance of the use of licensed products administered at correct dosages and the need for alternative molecules to avoid the onset of drug resistance phenomena.

## Figures and Tables

**Table 1:** Characteristics of the ornamental poultry farms, the breeds raised, and the pesticides used for the treatments against *D. gallinae*.

Farm Code	Region	Breeds	N° Chickens *	Pesticides Used **
**Cod_1a**	Piedmont	Silkie, Cocin, Polish	120	ByeMite^®^ (Phoxim) Neem Oil
**Cod_2a**	Piedmont	Orpington, Brahma	60–70	ByeMite^®^ (Phoxim)
**Cod_1b**	Emilia-Romagna	Orpington, Brahma	130	nrǂ
**Cod_2b**	Emilia-Romagna	Polish	180	nr
**Cod_1c**	Tuscany	Leghorn	100–110	nr
**Cod_2c**	Tuscany	Leghorn, Ancona	100	ByeMite^®^ (Phoxim) Neem Oil
**Cod_1d**	Basilicata	Ko-shamo, Cornish	40–60	ByeMite^®^ (Phoxim)
**Cod_2d**	Lazio	Silkie, Cocin, Cemani	250	ByeMite^®^ (Phoxim)
**Cod_1e**	Lombardy	Barnevelder	80–100	nr
**Cod_2e**	Lombardy	Faverolles, Cocin, Brahma	120–225	ByeMite^®^ (Phoxim) Neem Oil
**Cod_1f**	Sardinia	Silkie, Sebright	60	nr
**Cod_2f**	Umbria	Romagnola, Ancona, Polish	170	nr
**Cod_1g**	Marche	Barbuta d’Anversa	200–230	ByeMite^®^ (Phoxim)
**Cod_2g**	Veneto	Ermellinata di Rovigo	50–60	ByeMite^®^ (Phoxim)

* The number of chickens indicated refers to the average number or range of animals present on the farm during the course of the year (roosters and hens). ** The pesticides indicated refer to those declared by the farmers; ǂnr: not reported, the farmer does not report the use of pesticides. The possible use of fipronil is unknown.

**Table 2:** Lethality rates for each concentration and molecule.

Treatment	Concentration (ppm)	Lethality Rate (%)
**Fipronil**	500	99.8
	100	91.2
	20	72.4
	4	43.4
**Phoxim**	10,000	95.4
	2000	98.4
	400	93.1
	80	83.4
**Control**	-	22.2

**Table 3:** Comparison of mean lethality by treatment at the different concentration for fipronil and phoxim, respectively: 1st dilution: 500 and 10,000 ppm; 2nd 100 and 2000 ppm; 3rd 20 and 400 ppm; 4th 4 and 80 ppm.

	Treatment		
**Concentration**	**Fipronil**	**Phoxim**	***p-*** **Value**
**1st dilution**	0.998	0.954	*p* = 0.148
**2nd dilution**	0.912	0.984	*p* = 0.01
**3rd dilution**	0.724	0.931	*p* < 0.001
**4th dilution**	0.434	0.834	*p* = 0.001

## Data Availability

Data are contained within the article or Supplementary Materials.

## Supplementary Materials

The following supporting information can be downloaded at: https://www.mdpi.com/article/10.3390/vetsci9090486/s1. Table S1, raw data relating the assay results.
